# Prevalence and antibiogram of coagulase negative *Staphylococci* in bioaerosols from different indoors of a university in India

**DOI:** 10.1186/s12866-020-01875-8

**Published:** 2020-07-16

**Authors:** Himani Kumari, Trina Chakraborti, Madhuri Singh, Maneet Kumar Chakrawarti, Kasturi Mukhopadhyay

**Affiliations:** School of Environmental Sciences, Jawaharlal Nehru University, New Delhi, 110067 India

**Keywords:** Bioaerosol, Infectious bioaerosol, Staphylococci, CNS, Methicillin-resistance, Multi-drug resistance

## Abstract

**Background:**

Staphylococci species are the major constituents of infectious bioaerosols, particularly methicillin-resistant Staphylococci (MRS) have serious health impacts. Here, the bacterial burden was quantified, especially prevalence of MRS in bioaerosols collected from indoors of Dr. B.R. Ambedkar Central Library (DBRACL) and Central Laboratory Animal Resources (CLAR) of Jawaharlal Nehru University, New Delhi, India. Air samplings from DBRACL and CLAR were done using the settle plate method and SKC biosampler, respectively.

**Results:**

This study showed a maximum 6757 CFU/m^2^/hr of bacterial load in the DBRACL reading room, while unacceptable bacterial loads (> 1000 CFU/m^3^ of air) at different sites of CLAR. Further, at both the sampling sites the predominance of coagulase negative Staphylococci (CNS) was observed. A total 22 and 35 Staphylococci isolates were isolated from DBRACL and CLAR bioaerosols, respectively. Majority (16/22) of the Staphylococcal isolates from DBRACL belonged to human-associated Staphylococci where *S. haemolyticus* (5/22) was the most dominating species. However, in CLAR facility centre, animal-associated Staphylococci (19/35) were dominating, where *S. xylosus* (12/35) was the most dominating species. Further, antibiotic sensitivity tests revealed 41% MRS and 73% multidrug resistant (MDR) among airborne Staphylococci from DBRACL indoor bioaerosols. Similarly, in CLAR facility, approximately, 66% Staphylococci isolates were methicillin resistant, out of which 2 isolates showed high MIC value ≥ 16 μg/mL. Further, we confirmed the presence of 49% multidrug resistant Staphylococci in the indoor air of CLAR facility.

**Conclusions:**

This study suggested that the exposure of workers and students in CLAR to such a high concentration of drug-resistant Staphylococci should not be undermined, as these bacterial concentrations are the direct representative of inhalable particulate matter (PM_2.5_) as per collection procedure. Simultaneously, passive sampling from DBRACL assessed the risks due to microbial contamination in particle agglomerates, which may deposit on the crucial surfaces such as wounds/ cuts or on the frequently used items.

## Background

The increasing microbiological air pollution that is caused by the contaminated bioaerosols, has huge impact on the human health [1, 2]. Both the culturable and non-culturable bioaerosols can act as an important reservoir for antimicrobial resistance genes (ARG) by virtue of their free mobility, consequently putting people with compromised immunity at risk of catching infections [3–5]. In particular, the micro-organisms present in indoor bioaerosols are the direct threat to human health as they can cause infections with prolonged exposure [6, 7]. Therefore, checking the bacterial burden in indoor air has been frequently practiced in the hospital settings in order to mitigate the hospital-associated infections (HAIs) [3, 5, 6]. However, with the prevailing airborne antimicrobial resistance (AMR) and ARG in different environmental settings [4, 8, 9], the quantification of airborne indoor microbiome is a need of the present moment. Among airborne culturable bacteria, *Staphylococcus* species are the most common both in residential [10] as well as in hospital indoors bioaerosols [6, 11]. Staphylococci species are broadly divided into two groups: coagulase-positive Staphylococci (CPS such as *S. aureus*) and coagulase-negative Staphylococci (CNS such as *S. epidermidis*). Of total 47 Staphylococcal species, 7 (1 CPS and 6 CNS) belong to human-associated Staphylococci, which colonize specifically in humans, these are; *S. aureus*, *S. capitis*, *S. caprae*, *S. epidermidis*, *S. haemolyticus*, *S. hominis*, *S. lugdunensis*, *S. saprophyticus*, and *S. warneri* [12]. Among them, *S. aureus* is a leading cause of infections such as endocarditis, bacteremia, septic-shock. While *S. epidermidis*, *S. haemolyticus* and *S. saprophyticus* are CNS type, and are the most prevalent infective agents in hospital-acquired infections such as urinary tract infection (UTI) and indwelling medical device-associated bacteremia [12, 13]. Moreover, *S. aureus* is the most common human commensal and methicillin resistant *S. aureus* (MRSA) is commonly implicated in both hospital-acquired as well as in community-acquired infections Recently, CNSs are also reported as the most common bacterial pathogens in the hospital air [14–16]. Moreover, it is very difficult to eradicate CNS infections due to their species diversity leading to different anti-biograms and minimum inhibitory concentration (MIC) breakpoints [12, 17].

Previously the CNSs were considered as harmless bacteria but over the last few decades, treatment of CNS infections has become increasingly challenging due to the increasing number of methicillin resistant strains with reduced susceptibility to glycopeptides and other old and new antibiotics [18–20]. It was observed from previous studies that methicillin resistant CNS (MR-CNS) were dominating over methicillin-resistant *S. aureus* (MRSA) among clinical Staphylococci strains, the scenario continues till today [14, 19, 20].

Previous research regarding the prevalence of airborne total culturable bacteria in the household indoors has demonstrated that Staphylococci is dominant among airborne bacteria, and 66% of households were positive for MRSA with 2–80 CFU/m^3^ [10, 21]. Some confined environments in the university such as library and laboratory are the potential sites for the indoor bioaerosols sampling in order to evaluate the air quality inhaled by the students and staff residing there [22, 23]. Therefore, the present study was aimed to measure the total bacterial load in the bioaerosols of Dr. B.R. Ambedkar Central Library (DBRACL) and Central Laboratory Animal Resources (CLAR) environments in Jawaharlal Nehru University with special reference to airborne Staphylococci concentration. Further, the characterization of species diversity among collected Staphylococci was performed using both biochemical and molecular methods, followed by their antibiotic sensitivity profiling against commonly used anti-staphylococcal agents to estimate their multi-drug resistance level.

## Results

### Concentration of airborne bacteria and Staphylococci in Dr. B.R. Ambedkar central library (DBRACL) and central laboratory animal resources (CLAR)

**Fig. 1:**
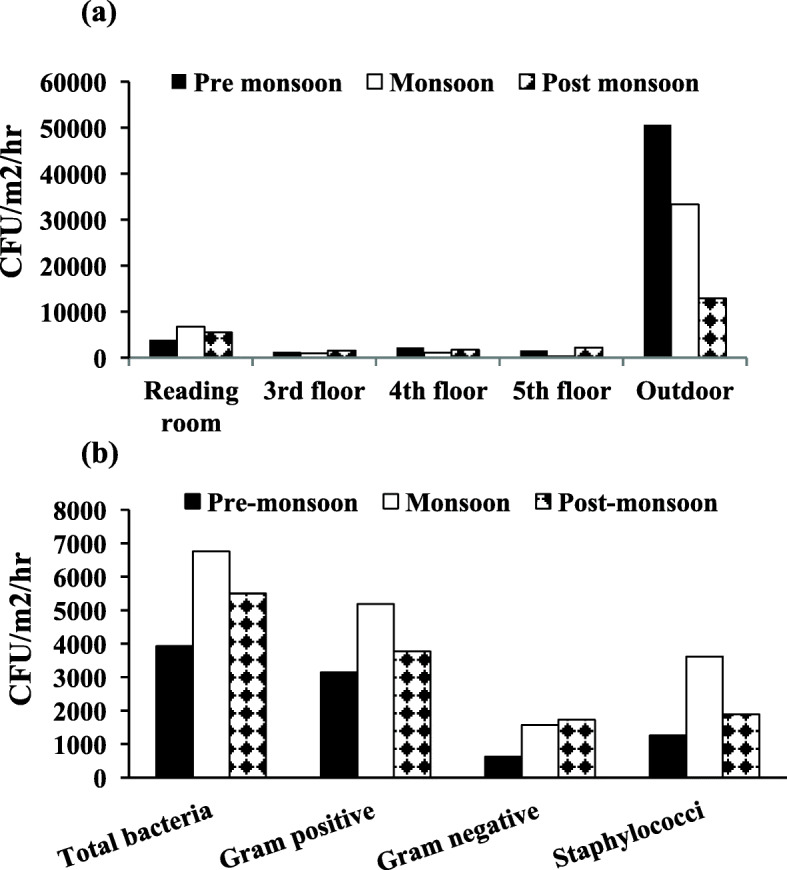
Bacterial loads in Dr. B.R. Ambedkar Central Library (DBRACL) Bioaerosols at different season: **a** Total bacterial load (CFU/m^2^/hr) in different rooms of the DBRACL; **b** Total bacteria, Gram positive bacteria, Gram negative bacteria and total *Staphylococci* in pre-monsoon, monsoon and post-monsoon in DBRACL reading room

In DBRACL the climatic conditions, temperature and relative humidity ranged between 27 °C–30.7 °C and 30.3–59%, respectively and the number of students in the reading room were varying from 96 to 112 during the entire course of air sampling. Overall, the bacterial counting was ranged from 314 to 6757 CFU/m^2^/h in DBRACL. Except for the reading room (on the ground floor), data from all other floors show low bacterial load (Fig. 1a). Total bacterial load in the reading room were found to be maximum (6756.76 CFU/m^2^/hr) during the monsoon season. However, bacterial load in the outdoor environment was extremely high i.e., 50,597 CFU/m^2^/hr, 33,312 CFU/m^2^/hr and 12884 CFU/m^2^/hr during the pre-monsoon, monsoon and post-monsoon, respectively (Fig. 1a). Therefore, further analyses were performed on the samples from Library reading room only.

Next, we characterized the bacterial load from reading room as Gram positive bacteria (GPB) and Gram negative bacteria (GNB) and compared their contribution in the total bacterial load (Fig. 1b). The data showed that the number of GPB was higher than that of GNB (Fig. 1b) in all seasons. As illustrated in the Fig. 1b, similar to total bacterial load, total GPB were also higher during the monsoon than pre-monsoon and post-monsoon. For example, in monsoon season GPB load was 5185.42 CFU/m^2^/hr, whereas in pre-monsoon and post-monsoon it was 3142.68 and 3771.21 CFU/m^2^/hr, respectively. In contrast, total GNB were higher during post-monsoon (1728.47 CFU/m^2^/hr) than pre-monsoon and monsoon (Fig. 1b). Like GPB, the Staphylococcal load in the reading room was found to be the maximum during monsoon sampling that was 3614.08 CFU/m^2^/hr (Fig. 1b). Since, the bacterial and staphylococcal load was observed maximum during monsoon in the university library, further, this study was extended to determine the bacterial and Staphylococci load in the indoor air collected from university CLAR during monsoon season.

**Fig. 2:**
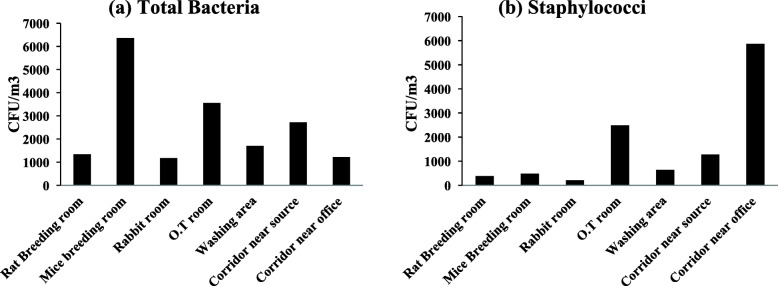
Prevalence of airborne **(a)** bacterial concentration and; **(b)** staphylococcal concentration (CFU/m^3^) in the indoor air of University Central Laboratory Animal Resources (CLAR)

A total load of culturable bacteria and Staphylococci was also very high in the CLAR indoors. A maximum total bacterial concentration was 6360 CFU/m^3^ in (Fig. 2a) in mice breeding room whereas Fig. 2b showed a maximum 5867 CFU/m^3^ airborne Staphylococcal concentration in the corridor near to the office. The minimum airborne total bacterial (1173 CFU/m^3^) and staphylococcal concentration (213 CFU/m^3^) was found in the rabbit room.

### Species diversity of airborne Staphylococci

**Fig. 3:**
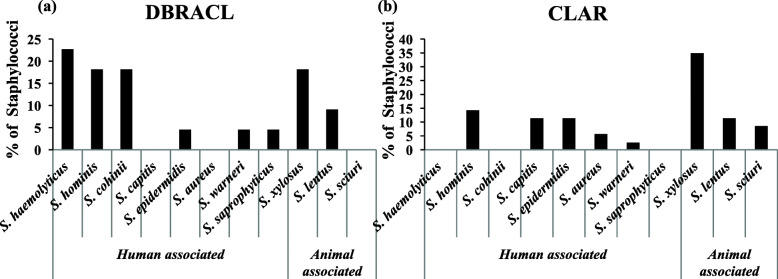
Prevalence of human-associated Staphylococci and animal-associated staphylococci in the University **(a)** DBRACL and **(b)** CLAR bioaerosols samples

A total of 22 *Staphylococci* strains were isolated from DBRACL reading room during this study. All of them were detected as CNSs by several biochemical tests as described in methods section. These 22 airborne *Staphylococci* from DBRACL were assigned to eight species of *Staphylococci*- *S. haemolyticus* (5/22)*, S. cohnii* (4/22)*, S. hominis* (4/22)*, S. epidermidis* (1/22), *S. warneri* (1/22), *S. saprophyticus* (1/22), *S. xylosus* (4/22) and *S. lentus* (2/22). Next, these staphylococcal isolates (16 out of 22) were characterized and confirmed by multiplex PCR [24], as well as by API Staph test [25] (Table 1 and Fig. 3a). The data revealed the dominance of human associated *Staphylococci* species (16/22 i.e., 72.7%) among these 22 CNSs (Fig. 3a), while the animal associated *Staphylococcus* species were only 27.2% (Fig. 3a). Moreover, *S. haemolyticus* (22.72%) was the most prevalent among human associated *Staphylococci*, while *S. xylosus* (18.18%) was the most prevalent among animal associated *Staphylococci* (Fig. 3a).

**Table 1:** Minimum inhibitory concentrations (MIC) of oxacillin (μg/mL) against staphylococcal strains isolated from DBRACL and CLAR bioaerosols

Sampling site	Staphylococci isolates		MIC of Oxacillin (μg/mL)	Susceptibility^a^	
				Susceptible (%)	Resistance (%)
**DBRACL**	**Human associated**	***S. haemolyticus*(*n*=5)**	**< 0.5-4**	**3 (60)**	**2 (40)**
		***S. cohnii*(*n*=4)**	**< 0.5-2**	**1 (25)**	**3 (75)**
		***S. hominis*(*n*=4)**	**< 0.5-16**	**3 (75)**	**1 (25)**
		***S. epidermidis*(*n*=1)**	**< 0.5**	**1 (100)**	**0 (0)**
		***S. warneri*(*n*=1)**	**< 0.5**	**1 (100)**	**0 (0)**
		***S. saprophyticus*(*n*=1)**	**1**	**0 (0)**	**1 (100)**
	**Animal associated**	***S. xylosus*(*n*=4)**	**< 0.5-1**	**2 (50)**	**2 (50)**
		***S. lentus*(*n*=2)**	**< 0.5**	**1 (50)**	**1 (50)**
**CLAR**	**Human associated**	***S. hominis*(*n*=5)**	**1-8**	**0 (0)**	**5 (100)**
		***S. capitis*(*n*=4)**	**<0.5-1**	**2 (50)**	**2 (50)**
		***S. epidermidis*(*n*=4)**	**<0.5-8**	**2 (50)**	**2 (50)**
		***S. aureus*(*n*=2)**	**1-8**	**1 (50)**	**1 (50)**
		***S. warneri*(*n*=1)**	**1**	**0 (0)**	**1 (100)**
	**Animal associated**	***S. xylosus*(*n*=12)**	**<0.5- 32**	**4 (33.3)**	**8 (66.7)**
		***S. lentus*(*n*=4)**	**<0.5-1**	**3 (75)**	**1 (25)**
		***S. sciuri*(*n*=3)**	**1**	**0 (0)**	**3 (100)**

^a^ For CNS: Susceptible (MIC ≤0.25 μg/mL); Resistant (MIC ≥0.5 μg/mL) and For *S. aureus*: Susceptible (MIC ≤2 μg/mL); Resistant (MIC ≥4 μg/mL)

In the university CLAR facility, 43 airborne coccus bacteria were obtained and among them 35 isolates were identified as Staphylococci. Out of 35 isolates of Staphylococci, 33 isolates were identified as CNS and 2 were as CPS i.e. *S. aureus*. These 35 staphylococcal isolates belonged to eight different species of *Staphylococcus*, namely; *S. xylosus* (12/35), *S. lentus* (4/35), *S. sciuri* (3/35), *S. hominis* (5/35)*, S. capitis* (4/35)*, S. epidermidis* (4/35)*, S. aureus* (2/35) and *S. warneri* (1/35) as presented in Table 1 (Fig. 3b). In sharp contrast to DBRACL, animal-associated Staphylococci (19/35) were in the majority among CLAR airborne staphylococcal isolates (Fig. 3b). Further, among detected animal-associated Staphylococci isolates, *S. xylosus* (35%) was the dominant species, while among human-associated Staphylococci isolates, *S. hominis* (14%) was the dominant species (Fig. 3b).

### Prevalence of methicillin-resistant Staphylococci in the indoor bioaerosols

Minimum inhibitory concentration (MIC) value of oxacillin of each staphylococcal isolate was determined by broth micro-dilution assay. According to CLSI interpretative criteria, *S. aureus* is considered as MRSA if oxacillin MIC is ≥ 4 μg/mL, CNS is considered as MR-CNS if MIC for oxacillin is ≥ 0.5 μg/mL [26] .

**Fig. 4:**
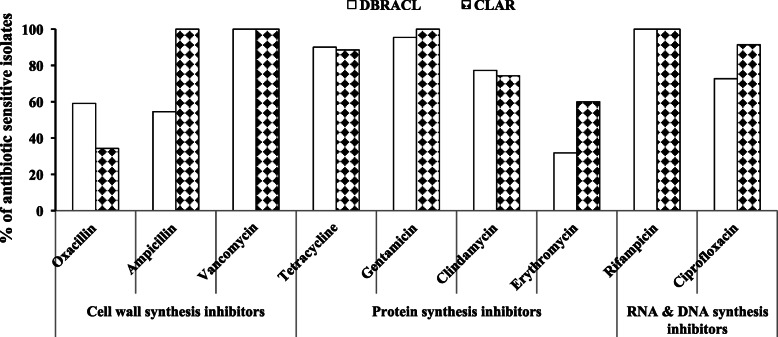
Overall sensitivity pattern of Staphylococci isolated from the bioaerosols collected at University DBRACL and CLAR against different classes of antibiotics

In DBRACL, MIC values of oxacillin were obtained ≤ 0.25 μg/mL for 13 CNS isolates, 0.5–2 μg/mL for 7 isolates and 4–16 μg/mL for 2 isolates (Table 1). Thus, the overall 59% of obtained isolates of CNSs were sensitive, while 41% were resistant to oxacillin (Fig. 4).

*S. haemolyticus* being the most dominant species among the isolated human-associated Staphylococci, showed MIC value ranging from < 0.5–4 μg/mL. Overall, 40% of *S. haemolyticus* (i.e., 2 out of 5) were methicillin (oxacillin) resistant. Among animal-associated, 50% isolates of *S. xylosus* with MIC value ranging from < 0.5–1 μg/mL showed resistance to oxacillin (Table 1).

In CLAR facility, overall, 22 out of 33 CNSs i.e., 67% were methicillin resistant CNS (MR-CNS). Among these 22 isolates, 2 showed high resistance i.e., ≥ 16 μg/mL. Among the most dominated animal associated CNSs, *S. xylosus* showed varying MIC value ranging from < 0.5 μg/mL to as high as 32 μg/mL. Moreover, ~ 67% (8/12) of *S. xylosus* isolates were methicillin resistant. Furthermore, 100% (total 5) isolates of *S. hominis* which was dominating species among human associated staphylococci, were oxacillin resistant with MIC value < 1–8 μg/mL (Table 1). Out of two *S. aureus* isolates one (i.e., 50%) was resistant to oxacillin with MIC value 8 μg/mL (Table 1).

Thus, similar to previous reports in literature [27, 28], this study also confirmed the > 40% prevalence of methicillin resistant Staphylococci in studied environment.

The *mecA* gene encoding for methicillin resistance in *S. aureus* has been widely distributed in CNS species, as demonstrated in previous studies from both India and abroad [29–31]. Therefore, further the occurrence of *mecA* gene was identified in all the CNS isolates obtained from two sites using *mecA* specific primers as described elsewhere [32]. However, in DBRACL, only one isolate was found *mecA* positive while in CLAR, none of the MR-CNS isolates harbored the *mecA* gene.

### Anti-biograms of airborne Staphylococci from indoor environments

Further the antibiotic-susceptibility spectrum of each Staphylococci isolate was determined against 9 different antibacterial agents from different classes (e.g., inhibitors of cell wall synthesis, protein synthesis, nucleic acid synthesis) including, oxacillin, vancomycin, tetracycline, ciprofloxacin, clindamycin, ampicillin, erythromycin, gentamicin and rifampicin (Fig. 4). Here, the proportion of resistant isolates is cumulative of resistant and intermediate value.

The MIC value of vancomycin and rifampicin against all the staphylococcal isolates obtained from both DBRACL and CLAR air was below their susceptibility breakpoints (CLSI guidelines 2009), indicating, 100% airborne CNS isolates were sensitive to these two last resort anti-MRSA agents (Fig. 4) [33]. The second most efficacious antibiotic was gentamicin for which 95 and 100% isolates from DBRACL and CLAR, respectively were sensitive. The third most active antibiotic was tetracycline, against which 90 and 89% isolates from DBRACL and CLAR, respectively were sensitive. Similarly, 72% DBRACL isolates and 91% CLAR isolates were ciprofloxacin sensitive. Furthermore, 77 and 74% clindamycin sensitivity was observed among environmental isolates from DBRACL and CLAR, respectively.

These isolates showed the highest resistance to erythromycin and oxacillin. For example 68% DBRACL isolates, 40% of CLAR isolates were erythromycin resistant. Likewise, 41 and 66% of CNS isolates were oxacillin resistant in DBRACL and CLAR, indoor air, respectively. In contrast to oxacillin, CNS isolates were more susceptible towards ampicillin with 45 and 0% ampicillin resistant isolates detected from DBRACL and CLAR facilities, respectively. This may be due to the discontinuity of ampicillin usage in the clinical settings, thereby reducing the rate of evolution of ampicillin resistant mutants in the absence of antibiotic in environment. However, approximately 90.9% isolates from DBRACL (i.e., 20/22) were either resistant or intermediate to the one or more antibiotics.

Overall, these airborne staphylococcal isolates showed resistance to the most common and frequently used conventional antibiotics such as oxacillin and erythromycin.

### Multidrug resistance profile of airborne Staphylococci

So far, oxacillin has not been the effective antibiotic since most of the isolates were resistant to it. Next, multidrug resistance profile of each isolate from both DBRACL and CLAR aerosols was analyzed. MIC value of all the multidrug resistant (resistant to ≥ 2 drugs) staphylococcal strains belonging to different species are presented in Table 2 and Table 3 for DBRACL and CLAR samples, respectively.

**Table 2:** MIC of antibiotics against Staphylococcal isolates of those species, which showed multidrug resistance in the DBRACL

***Staphylococci*** isolates		Strain ID	Antibiotics^a^						
			Minimum inhibitory concentrations (μg/mL)						
			Oxa	Tet	Ery	Clin	Cip	Amp	Genta
**Human associated**	***S. haemolyticus***	**S24**	**1 (R)**	**≤ 0.5(S)**	**>32 (R)**	**< 0.0625(S)**	**0.5(S)**	**0.5 (R)**	**< 0.0625(S)**
		**S41**	**0.25(S)**	**16 (R)**	**32 (R)**	**4 (R)**	**>32 (R)**	**8 (R)**	**0.25(S)**
		**S43**	**0.5 (R)**	**≤ 0.5(S)**	**0.25(S)**	**4 (R)**	**0.5(S)**	**0.25(S)**	**0.125(S)**
		**S53**	**4 (R)**	**16 (R)**	**0.5(S)**	**4 (R)**	**0.5(S)**	**>32 (R)**	**< 0.5(S)**
	***S. cohnii***	**S6**	**0.5 (R)**	**≤ 0.5(S)**	**>32 (R)**	**>32 (R)**	**1(S)**	**0.25(S)**	**< 0.5(S)**
		**S14**	**0.25(S)**	**≤ 0.5(S)**	**64 (R)**	**>32 (R)**	**0.5(S)**	**0.25(S)**	**< 0.0625(S)**
		**S22**	**0.5 (R)**	**≤ 0.5(S)**	**8 (R)**	**0.25(S)**	**32 (R)**	**1 (R)**	**< 0.0625(S)**
	***S. hominis***	**S21**	**0.125(S)**	**≤ 0.5(S)**	**32 (R)**	**< 0.0625(S)**	**1(S)**	**0.25(S)**	**16 (R)**
		**S45**	**16 (R)**	**≤ 0.5(S)**	**8 (R)**	**< 0.0625(S)**	**>32 (R))**	**0.5 (R)**	**< 0.5(S)**
	***S. epidermidis***	**S52**	**0.25(S)**	**≤ 0.5(S)**	**>32 (R)**	**< 0.0625(S)**	**0.25(S)**	**4 (R)**	**< 0.5(S)**
**Animal associated**	***S. xylosus***	**S17**	**0.5 (R)**	**≤ 0.5(S)**	**>32 (R)**	**< 0.0625(S)**	**0.25(S)**	**0.125(S)**	**< 0.5(S)**
		**S27**	**0.125(S)**	**≤ 0.5(S)**	**32 (R)**	**< 0.0625(S)**	**1(S)**	**8 (R)**	**0.5(S)**
		**S37**	**0.125(S)**	**≤ 0.5(S)**	**8 (R)**	**< 0.0625(S)**	**0.25(S)**	**2 (R)**	**1(S)**
		**S47**	**2 (R)**	**≤ 0.5(S)**	**1 (I)**	**< 0.0625(S)**	**>32 (R)**	**16 (R)**	**0.125(S)**
	***S. lentus***	**S15**	**0.5 (R)**	**≤ 0.5(S)**	**0.25(S)**	**0.25(S)**	**>32 (R)**	**0.25(S)**	**0.0625(S)**
		**S49**	**0.25(S)**	**≤ 0.5(S)**	**>32 (R)**	**0.125(S)**	**0.25(S)**	**0.5 (R)**	**< 0.0625(S)**

^a^*Oxa* Oxacillin, *Tet* Tetracycline, *Ery* Erythromycin, *Clin* Clindamycin, *Cip* Ciprofloxacin, *Amp* Ampicillin, *Genta* Gentamicin, *S* Sensitive, *I* Intermediate, *R* Resistant

**Table 3:** MIC of seven antibiotics against Staphylococcal isolates of those species which showed multidrug resistance in CLAR

***Staphylococci*** isolates		Strain ID	Antibiotics^a^						
			Minimum inhibitory concentrations (μg/mL)						
			Oxa	Tet	Ery	Clin	Cip	Ceph	Amo
**Animal associated**	***S. xylosus***	**AHOTR13**	**32 (R)**	**0.5 (S)**	**2 (I)**	**0.5(S)**	**0.25(S)**	**4(I)**	**0.25(S)**
		**AHRR2**	**0.25(S)**	**0.25(S)**	**0.13(S)**	**4(R)**	**0.5(S)**	**0.03(S)**	**0.5(I)**
		**AHRBR4**	**8 (R)**	**32 (R)**	**0.13 (S)**	**0.5 (S)**	**0.5 (S)**	**4(I)**	**0.25(S)**
		**AHCRNS7**	**1 (R)**	**32 (R)**	**4 (I)**	**0.5 (S)**	**0.25 (S)**	**4(I)**	**0.25(S)**
		**AHWR4**	**1 (R)**	**0.5 (S)**	**32 (R)**	**1 (I)**	**0.25 (S)**	**2(S)**	**0.5(I)**
	***S. lentus***	**AHCRNS2**	**<0.5(S)**	**0.5(S)**	**0.25(S)**	**1(I)**	**0.25(S)**	**4(I)**	**0.06(S)**
		**AHCRNS3**	**<0.5(S)**	**0.5(S)**	**0.25(S)**	**1(I)**	**0.25(S)**	**4(I)**	**0.06(S)**
		**AHCRNS20**	**<0.5(S)**	**0.5(S)**	**1(I)**	**1(I)**	**0.25(S)**	**0.5(S)**	**0.03(S)**
		**AHCRNS21**	**1(R)**	**0.5(S)**	**4(I)**	**1(I)**	**0.25(S)**	**0.25(S)**	**0.03(S)**
	***S. sciuri***	**AHRR1**	**1 (R)**	**0.5 (S)**	**2 (I)**	**4 (R)**	**4 (R)**	**0.03(S)**	**0.25(S)**
		**AHCRNS10**	**1 (R)**	**8(I)**	**2 (I)**	**2(I)**	**1(S)**	**1(S)**	**0.13(S)**
		**AHWR3**	**1 (R)**	**0.5 (S)**	**32 (R)**	**1 (I)**	**0.25 (S)**	**2(S)**	**0.5(I)**
**Human associated**	***S. hominis***	**AHCRNO5**	**1(R)**	**1(S)**	**4(I)**	**0.06(S)**	**0.25(S)**	**4(I)**	**0.25(S)**
		**AHCRNO11**	**4(R)**	**2(S)**	**1(I)**	**0.25(S)**	**1(S)**	**1(S)**	**0.25(S)**
		**AHWR6**	**8(R)**	**0.5(S)**	**2(I)**	**0.25(S)**	**1(S)**	**1(S)**	**0.25(S)**
	***S. capitis***	**AHCRNS12**	**1(R)**	**8(I)**	**1(I)**	**0.13(S)**	**2(I)**	**1(S)**	**0.25(S)**
	***S. epidermidis***	**AHMBR4**	**8(R)**	**4(S)**	**1(I)**	**0.25(S)**	**0.06(S)**	**0.13(S)**	**0.5(I)**

^a^*Oxa* Oxacillin, *Tet* Tetracycline, *Ery* Erythromycin, *Clin* Clindamycin, *Cip* Ciprofloxacin, *Amo* Amoxicillin, *Ceph* Cephalothin, *S* Sensitive, *I* Intermediate, *R* Resistant

In DBRACL, approximately 73% staphylococcal isolates (i.e., 16/22) showed resistance to two or more antibiotics (Table 2), majority of the isolates were resistant to erythromycin followed by oxacillin and ampicillin. Particularly, the multidrug resistance pattern was exhibited by six species; *S. haemolyticus*, *S. cohnii, S. hominis, S. epidermidis, S. xylosus,* and *S. lentus.* Notably, some isolates of *S. haemolyticus* were resistant to five antibiotics.

Of note, all the isolates belonging to some of the staphylococcal species from library indoor air such as *S. cohnii, S. epidermidis, S. xylosus* and *S. lentus*, showed multi-drug resistance.

While in CLAR facility, there were at least 17 isolates out of 35 (i.e., 48.57%) belonging to different *Staphylococcus* species, which were resistant to two or more drugs. Moreover, two isolates of *S. xylosus,* all the three isolates of *S. sciuri* and one isolate of *S. capitis* were resistant to four antimicrobial drugs. Overall, 5 out of 12 (i.e., 41.67%) isolates of *S. xylosus,* 3 out of 5 (i.e., 60%) isolates of *S. hominis*, 1 out of 4 (i.e., 25%) isolate of *S. capitis* and *S. epidermidis* exhibited multi-drug resistant pattern, while, all the strains (i.e., 100%) of *S. lentus* and *S. sciuri* showed multi-drug resistance. Furthermore, 6 of total 12 animal associated Staphylococci isolates showed resistance to both erythromycin and clindamycin (Table 3).

## Discussion

Indoor air quality is getting deteriorated due to the microbiological contamination of bioaerosols, which could expose the inhabitants, particularly those with compromised immunity are at high risk due to potential airborne pathogens.

*Staphylococcus* is one of the dominant genera among indoor airborne microbiomes both in hospital and other residential buildings [21, 34]. The spread of multidrug resistant Staphylococci in environment from different sources has already been reported in India [30, 35–37]. Furthermore, Staphylococci species such as MRSA and MR-CNSs are the biggest contributors in the spread of antibiotic resistant genes (ARGs) in the environment as reported from India and as well as from abroad [3–5, 9, 38]. CNSs are the common microflora of human skin, however, they can be pathogenic and virulent if they colonizes the open wounds. Of late, MR-CNSs are commonly prevalent in indoor/outdoor environments of hospitals and communities [13, 39, 40].

Apart from frequently associated with nosocomial infections, these infectious agents are also of great concern in a variety of indoor environments such as libraries, offices and other residential indoors [10, 34]. Although, we have adequate knowledge of MRSA epidemiology and its role in spreading antimicrobial resistance (AMR) in the environment, there is comparatively scant knowledge of MR-CNS epidemiology in indoor bioaerosols and its AMR pattern in India, which is imperative to evaluate the indoor air quality [9, 11, 21, 22, 41]. Lately, multi-drug resistant CNSs have been dominating in all kinds of microbiomes whether it is environmental samples, hospital-wastes, poultry, agricultural farms etc., thereby they are equally clinically relevant [15, 36, 42]. Recent study in India based on bioaerosols also showed the abundance of methicilin resistant Staphylococci in residential buildings in central India [37]. Yadav et al., have studied the abundance of bacterial and fungal loads in different residential indoor environments near and far from the Gwalior trade fair site [30]. They showed the higher presence of MR-CNS in bioaerosols from trade fair ground, compared to non-fair sites during the fair event [30]. Exploring the microbial contamination of indoor bioaerosols in libraries and experimental areas, where students spend a significant time would be very relevant from their health’s perspective [41]. Therefore, the present study was focused on the evaluation of the prevalence of Staphylococci in the indoor air of the University central library (DBRACL) and animal research facility (CLAR), followed by their species characterization and antibiotic sensitivity screening.

In DBRACL, we recorded as much as 6757 CFU/m^2^/hr, which was the highest concentration of airborne bacteria including staphylococci from the reading room during monsoon season (Fig. 1a and b), perhaps due to the optimal growth conditions for microorganisms are present during this time. Reasonably, the reading room was highly dense, compared to 3rd floor, 4th floor and 5th floor study areas, which are dedicated to a specific study area (see material and methods). Furthermore, we observed that the numbers of GPB were always higher than the number of GNB (Fig. 1b), indicating that the GPB were predominantly present in the DBRACL air. These findings are similar to the previous report by Di Giulio et al., where GPB was detected in majority in the indoor bioaerosols of a university of Italy [41]. Of note, air sampling from DBRACL reading room using the settle plate method gives an idea of viable microbial contaminants in the dust, which can deposit over the frequently touched surfaces and may cause potential human health implications after coming into contact [43].

Subsequently, we isolated 22 Staphylococci strains from DBRACL reading room. Most striking observation was that all the 22 Staphylococci strains isolated from DBRACL indoors were identified as CNSs (Fig. 3a and Table 1). Furthermore, 16 out of the 22 were human-associated CNSs, suggesting that humans are the likely source of airborne Staphylococci in the library, which is quite reasonable as only the students and university staff are permitted in the library. Furthermore, *S. haemolyticus* was the most prevalent human-associated CNS, followed by *S. cohnii* and *S. hominis*, among airborne Staphylococci from library indoors. Similar to this observation, previously also *S. haemolyticus* had been found the most frequently isolated CNS in hospital environments, after *S. epidermidis* from the clinical samples, and is a newly emerging aetiological agent of infectious diseases [13, 40]. Based on the oxacillin MIC value, 41% of airborne Staphylococci were methicillin resistant-CNS (MR-CNS). Of late, the CNS has been frequently implicated in HAIs as well as in the community acquired infections (CAIs) in Indian continent [36, 44]. Further, antibiotic resistance profiling of these airborne CNSs from DBRACL indoors demonstrated 50% of Staphylococci were ampicillin resistant, 73% were erythromycin resistant, 27% were ciprofloxacin resistant, 23% were clindamycin resistant, 9% were tetracycline resistant and 5% were gentamicin resistant. Our study demonstrated 73% of CNSs were resistance to more than two antibiotics from DBRACL indoor bioaerosols. Moreover, CNSs are the frequent cause of Staphylococcal infections both in human and animals, and they tend more to develop multi-drug resistance [19, 45].

Using the active sampling in CLAR facility, we evaluated an inhalable exposure of humans and animals to microbial contamination, where we observed an unacceptable exposure (≥ 1100 to as much as 6360 CFU/m^3^) of total airborne bacteria at all the indoor locations of animal facility (Fig. 2a). Likewise, the Staphylococci load was also high at most of the indoor locations of the animal facility of university (Fig. 2b). Nevertheless, similar to the library, a predominance of CNS species was observed in the animal facility of the university. Out of total 35 isolates of Staphylococci from CLAR indoors, 33 were CNSs, which were classified into 8 different species (Fig. 3b). As expected, in contrast to the DBRACL, the majority (54%) of CNS species from CLAR were animal-associated, these were; *S. xylosus* (12/33), *S. lentus* (4/33) and *S. sciuri* (3/33). While, human associated Staphylococci species from CLAR were, *S. hominis, S. capitis, S. epidermidis* and *S. aureus*. Notably*, S. xyloxus* was the most frequently found animal associated Staphylococci species from CLAR, which is also commonly reported in the genital tract of rabbit and in various other animals such as mouse, rat, chicken and swine [46, 47]. Whereas *S. hominis* was the dominant species among human-associated Staphylococci from CLAR indoors. The animal associated species *S. lentus* and *S. sciuri* are also the inhabitant of mouse, rat, swine and chicken [47]. Since, CLAR has laboratory animals (mice, rat and rabbit) for experimental purposes; they may be the most likely source of these dominating animal-associated Staphylococci in there indoors.

Further, the presence of ~ 66% of CNS species were MR-CNS (Fig. 4) and 49% of CNS species were multi-drug resistant (MDR) in the indoor air of CLAR. *S. xylosus* being the highest prevalent airborne bacteria in CLAR, showed resistance to a wide variety of antimicrobial drugs used in this study. A similar multi-drug resistance pattern of *S. xylosus* isolates from chicken barn bioaerosols was reported elsewhere [48]. Furthermore, all the isolates of *S. lentus* and *S. sciuri* showed multi-drug resistance pattern. Moreover, isolates from 6 CNS species out of total 8 different CNS species obtained were resistant to the two or more drugs, which were characterized as multi-drug resistant (Table 3). Our study is supporting the previous reports about the predominance of CNS species in environmental samples, and their increasing resistance to the existing conventional antibiotics [19, 40, 45].

These MDR Staphylococci were found to be resistant to the conventional antibiotics such as erythromycin, clindamycin, tetracycline and ciprofloxacin. On the contrary, the old and abandoned beta-lactam antibiotic ampicillin was found to be very effective, which may be because of the absence of any selective pressure due to its decreasing use.

A recent study by May et al. showed increased rates of resistance to oxacillin and high rates of multidrug resistance among CNS species from USA over the past decade [49]. Out of total 57 CNSs (22 from library and 35 from CLAR), *mecA* gene was detected in only one MR-CNS strain (identified as *S. hominis*) from the library, which showed a high MIC (16 μg/mL) of oxacillin. Out of total airborne MR-CNSs, we only found 2 strains with MIC > 8 μg/mL, one strain with oxacillin MIC of 16 μg/mL from DBRACL, which was also detected as *mecA* positive, and another with oxacillin MIC of 32 μg/mL from CLAR, which was *mecA* negative. Thus, resistance in the *mecA* negative MR-CNSs might be due to some other mechanisms; 1) overproduction of the beta-lactamase, which inactivates the oxacillin [50, 51], 2) secretion of modified penicillin binding protein (PBP) with reduced affinity to beta-lactam drugs [52], 3) increase in the PBP expression [53]. Mostly, these airborne *mecA* negative MR-CNS strains may be categorized in the borderline methicillin resistance phenotype, which exhibits low-level of oxacillin resistance with MIC value < 8 μg/mL. However, in this study we used only single PCR method to identify the *mecA* gene, therefore, the absence of other *mec* type genes in these isolated airborne MR-CNSs could not be excluded. Instead, the use of multiplex PCR as previously described [54] is required to fully identify all *SCCmec* type genes which will be pursued in future.

A similar non-linkage between *mecA* genotype and methicillin resistance phenotype has also been reported previously both in MR-CNS [50] and MRSA strains [55]. The ability of CNS to act as reservoir for resistance genes and to transfer them into other pathogens such as *S. aureus* is other important dimension to the problem of antibiotic resistance. As shown previously that the bacteria obtained from animals and animal dwelling places are the major cause for the spreading of pathogens to other animals as well as humans [38]. Moreover, Staphylococci species are the rapidly emerging threat to human health, particularly CNSs are largely implicated in skin and soft tissue infections (SSTIs) such as in patients with burns and have undergone surgeries [12, 20]. CNS species frequently found responsible for infecting wounds and medical implants inside a hospital include: *Staphylococcus capiti, Staphylococcus haemolyticus, Staphylococcus epidermidis*, *Staphylococcus lugdunensis*, *Staphylococcus hominis*, *Staphylococcus saprophyticus*, *Staphylococcus simulans* and *Staphylococcus auricularis* [39, 56].

This study also recommends that the exposure of workers and students in CLAR to such a high concentration of Staphylococci (5866 CFU/m^3^) beyond the acceptable limit of microbial exposure as described in WHO guidelines [57], should not be undermined, as this SKC based active collection method captures the inhalable particulate matter (PM_2.5_) with up to 90% efficiency. However, the actual level of microbial contamination is likely to be higher in CLAR if the bioaerosols containing larger size particulate matters including PM_10_ and agglomerates were examined. The larger size bioaerosols can also settle down on the external body surfaces resulting in the colonization of the skin and soft tissues and particularly vulnerable for cuts and wounds**.** Evidently, some of the CNS species such as *S. epidermidis*, *S. hominis* and *S. hemolyticus* etc., detected here from both the sites (Fig. 3a-b) have already been implicated in SSTIs [56], these may cause potential health risks in humans having open wounds or may also alter the results in animal studies where surgeries are employed.

On the other hand, the settle plate method only reflects the bacterial burden in large dust particles including agglomerates, therefore, an estimation of inhalable exposure of humans in the library to microbial contaminants was not possible. At the same time this passive sampling gives us a risk assessment of the contaminated air if it deposits onto a crucial surfaces such as wounds/ cuts or on the frequently used items in the library environment such as books, mobiles, laptops etc. [43, 56].

## Conclusions

Overall, this study identified that both the facilities contained an unacceptable airborne Staphylococcal loads with a huge proportion of MR-CNS and MDR-CNS, which could affect human health, especially people with compromised immunity. Most notably, a majority of the airborne MR-CNS identified in this study may be categorized under low-level methicillin resistance phenotype. However, the major limitation of this study is the use of two different sampling methods, for which the bacterial burden could not be directly compared between two sites. Nevertheless, this study provided an efficient estimation of airborne staphylococcal species diversity and their antibiogram in the indoor environments, which might be helpful in deciding the strategy for the successful eradication of MDR-CNSs.

## Methods

### Description of sampling sites

#### Dr. B.R. Ambedkar central library (DBRACL) of Jawaharlal Nehru University

Bioaerosols sampling to evaluate bacterial load in indoor environment was carried out in DBRACL of Jawaharlal Nehru University. It is a 40 year old, nine storied building situated in the central part of the campus where thousands of students studying every day and night. The building comprises of a huge reading hall on the ground floor and other eight floors with books arranged in different sections based on subjects. Along with the main reading hall (ground floor), three other sections namely, Science section (3rd floor), European language section (4th floor) and Afro-Asian language section (5th floor) were selected randomly for the air sampling as they have similar pattern of book storage.

#### Central laboratory animal resources (CLAR) of Jawaharlal Nehru University

The air sampling was carried out during monsoon in the CLAR facility of Jawaharlal Nehru University, New Delhi, India. CLAR is a two-storied building, situated in the south corner of the campus. It comprises of three different areas viz., the animal rooms (rat breeding room, mice breeding room, operation theatre and rabbit room), corridors and washing area. This building has heating ventilating and air conditioning (HVAC) ventilation system. CLAR has limited human interference except for the staff and visiting research students for laboratory experiments.

#### Air sampling by the settle plate technique

In DBRACL, air samplings were done in the first week of pre-monsoon (May), monsoon (July) and post-monsoon (September) in 2015 by settle plate technique using non-selective nutrient rich Brain heart infusion (BHI) media. 9 cm petriplate containing appropriate growth medium was exposed to air with open lid by keeping it at 1–1.5 m above the ground for 1 hr. Cycloheximide (0.2 mg/mL) was added in the media to inhibit the fungal growth [43]. Relative humidity (RH) and temperature were also recorded with the help of Mextech TM-1 Thermo Hygro digital clock (Mextech technologies, India) at the time of sampling. Relative humidity varied from 30.30 to 39%, 36.66 to 63% and 38 to 51% during pre-monsoon, monsoon and post-monsoon, respectively. The temperature varied from 27.9 °C to 33.1 °C, 28.6 °C to 30.7 °C and 27 °C to 31.5 °C during pre-monsoon, monsoon and post-monsoon, respectively. While the wind speed was still in the reading room indoor due to fully air-conditioned, it was approximately 0.5 mph, while rest of the locations were naturally ventilated. The sampling was also carried out in the outdoor air around the library, the climatic conditions for the same have been included in Table 4.

**Table 4:** Climatic conditions at DBRACL and CLAR during air sampling in 2015

Sampling site	Sampling place	Pre-monsoon (May)		Monsoon (July)		Post-monsoon (September)		Type of ventilation
		Temperature (°C)	Relative humidity (%)	Temperature (°C)	Relative humidity (%)	Temperature (°C)	Relative humidity (%)	
**DBRACL**	**Reading room**	**28.5**	**31.3**	**28.6**	**36.7**	**27**	**38**	**HVAC**
	**Third floor**	**27.9**	**39**	**29.4**	**59**	**30**	**42**	**Natural**
	**Fourth floor**	**29.4**	**30.3**	**29.7**	**58**	**29.6**	**46**	**Natural**
	**Fifth floor**	**30.7**	**30.6**	**29.5**	**58**	**29.7**	**51**	**Natural**
	**Outdoor**	**33.1**	**34**	**30.7**	**63**	**31.5**	**38**	**Natural**
**CLAR**	**Rat Breeding room**	**Not** **collected**		**25.6**	**41**	**Not** **collected**		**HVAC**
	**Mice Breeding room**			**24.8**	**42**			**HVAC**
	**Rabbit room**			**21.5**	**65**			**HVAC**
	**Washing place**			**26.8**	**90**			**Natural**
	**O.T room**			**23.7**	**73**			**HVAC**
	**Corridor near source area**			**23.3**	**44**			**HVAC**
	**Corridor near office**			**25.9**	**91**			**Natural**

Footnote: Heating, ventilating and air conditioning (HVAC)

All equipment and materials were handled aseptically to ensure that the samples were not contaminated. After bioaerosols collection, plates were transferred to the lab (which is at 5 min distance from the sampling site) on ice in a UV sterile carbon box, followed by incubation at 37 °C for 48 h for bacterial colony growth.

#### Active sampling using SKC biosampler

In CLAR facility, air samples were collected during monsoon in July 2015 by using SKC biosampler (Cat. 225–9594, SKC, UK) at the flow rate of 12.5 L/min for 30 min (recommended sampling time by the manufacturer for water based collecting liquids) in 20 ml of 10 mM phosphate buffer saline (PBS) (pH 7.4). This SKC biosampler, resembles an All-glass Impinger (AGI-30), collection efficiency is close to 100% over a wide range of particle sizes when operated at 12.5 L/min with water and liquid of similar viscosity. For particle < 1.0 μm diameter, collection efficiency decreased to approximately 90% at 0.5 μm. Thus, this SKC based active collection method captures the inhalable particulate matter (PM_2.5_) with 100% efficiency [58]. Temperature and RH were also recorded during air sampling (Table 4). Temperature varied from 21.5 °C to 26.8 °C while RH varied from 41 to 91%. All equipment and materials were sterilized before sampling to ensure that the collected air samples were not contaminated.

After sampling, 0.1 mL aliquots and their 10 fold dilutions were spread onto BHI agar plate for total airborne bacterial enumeration and onto mannitol salt agar (MSA) plate for airborne Staphylococci counting. MSA was used in order to differentiate CPS and CNS [59].

#### Enumeration and isolation of bacterial colonies

The bacterial loads obtained on 9 cm petri plate exposed for 1 hr, were expressed as CFU/m^2^/hr [43]. After incubation, plates were counted for total culturable bacteria and then refrigerated at 4 °C until further analyses. For characterization of bacteria, we transferred each colony aseptically from primary plate to secondary plates containing a selective medium, for example Eosin methylene blue (EMB) for isolation of GNB, Sheep Blood agar (BA) media plates for GPB and MSA for Staphylococci. All the media were purchased from HiMedia, India. After secondary plating in these selective media, plates were incubated for 24 hr at 37 °C. Total GPB, GNB and staphylococci were counted after the 48 hr incubation.

#### Characterization of Staphylococci

Bacterial colonies grown on MSA plate were further processed for colony morphology by Gram staining, followed by biochemical tests such as catalase test, MSA test, coagulase test, and staphylo monotec test kit plus (Fluka, Sigma-Aldrich) for confirmatory test of *S. aureus*. Oxidase test (Sigma-Aldrich) was done for CNS. *Staphylococcus* species showed as Gram positive cocci in clusters under microscope after Gram staining. In MSA test *S. aureus* ferments mannitol, lowering down the pH of the media, phenol red indicator turns yellow and thus formed yellow colored colonies on MSA agar plate whereas CNS colonies were pink in color on MSA plates [60]. *S. aureus* were further characterized based on their colony morphology using Gram staining, followed by confirmatory biochemical tests such as oxidase test, catalase test, coagulase test and staphylo-monotec test (Fluka, Sigma-Aldrich). Staphylococci show negative result for oxidase test. There are certain bacteria, which have ability to produce colored compound indophenol blue from the oxidation of N, N-dimethyl-p-phenylenediamine oxalate and α-naphthol in the presence of the enzyme cytochrome oxidase. For oxidase test, bacterial smear was spread over oxidase disc containing N, N-dimethyl-p-phenylenediamine oxalate and which were found to be negative as it does not change the color of the disc [60] . Catalase is an antioxidant enzyme that is produced by aerobic microbes in order to neutralize toxic hydrogen peroxide (H_2_O_2;_ an oxygen metabolite). This enzyme decomposes the H_2_O_2_ to water and oxygen and thus resulting in rapid oxygen bubble production. For catalase test 3% H_2_O_2_ was applied on bacterial smear and immediate oxygen bubbling was produced. Staphylococci give positive catalase test. Coagulase test is used to differentiate *S. aureus* from CNS. *S. aureus* produces coagulase enzyme which converts soluble fibrinogen to insoluble fibrin present in plasma, thus agglutinating the blood cells. Coagulase tests were performed using coagulase plasma from rabbit (HiMedia, India). *S. aureus* gives positive coagulase test. Further, monotec test was performed to differentiate between *S. aureus* and other Staphylococci species of CPS class. *S. aureus* gives positive monotec test. Staphylo-Monotec test kit plus (Fluka, Sigma-Aldrich) was used for this purpose. The test reagent consists of monodisperse particles, which are coated with fibrinogen and immunoglobulin G (IgG). *S. aureus* has protein A in its cell wall. Fibrinogen binds to the coagulase which is a cell associated enzyme, and the Fc part of immunoglobulin G binds to protein A. When *S. aureus* is mixed with the Staphylo-monotec test reagent, a rapid agglutination occurs as a result of binding of fibrinogen to coagulase enzyme and IgG to protein A. After identification, bacterial isolates were stored in 30% glycerol at − 80 °C for further analyses.

Finally, from the air of the reading room of the library, after repeated sub-culturing on MSA plates and subsequently confirmed by API staph kit, we could identify total 22 Staphylococci strains.

In a similar fashion after active sampling from CLAR, 35 colonies from MSA plates were confirmed as Staphylococci strains.

#### Antibiotic susceptibility testing of staphylococcal isolates

Antibiotic susceptibility of all the airborne staphylococcal isolates were determined by measuring minimum inhibitory concentration (MIC) in cation adjusted-Mueller Hinton Broth (MHB) by broth micro-dilution method according to the Clinical Laboratory Standard Institute guideline [26]. The MIC is defined as the lowest drug concentration preventing visible turbidity after 24 h of incubation at 37 °C. A final concentration of inoculums of 10^5^ CFU/mL concentration of bacterial culture was used to determine MICs. We determined the MICs of oxacillin against all collected staphylococcal isolates. *S. aureus* ATCC 33591, *S. aureus* ATCC 29213 and *S. epidermidis* ATCC 35984 were used as reference strains during determination of MICs, as well as during each confirmatory biochemical tests. The MIC breakpoints of oxacillin against *S. aureus* are ≥4 and ≤ 2 μg/mL for resistant and susceptible strains, respectively. Against CNS, MIC breakpoints of oxacillin are ≥0.5 and ≤ 0.25 μg/mL for resistant and susceptible strains, respectively [26]. The group of CNS isolates which showed susceptibility and resistance to oxacillin, were considered as MS-CNS and MR-CNS, respectively and were further tested against eight antibiotics of different class, these are vancomycin, tetracycline, rifampicin, ciprofloxacin, clindamycin, ampicillin, erythromycin and gentamicin. The multidrug resistance in bacteria is defined when an organism is resistant to at least two classes of antibiotics [61]. Susceptibility breakpoint for each antibiotic was followed as per CLSI guidelines [26].

#### Species level identification of environmental Staphylococci isolates

All the staphylococci isolates including both CPS and CNS were further identified at species level by commercial identification system API–staph test kit (BioMerieux, France) [25], according to manufacturer’s guidelines. Briefly, purified staphylococci isolates were grown on BHI agar for 18–24 hr, a homogeneous mixture of each isolate was prepared by suspending colonies in staph-medium (provided with the kit) with turbidity corresponding to 0.5 Macfarland standard. This suspension was used to fill the microtubes of API-strips, followed by strip incubation at 37 °C for 24 hr. After incubation, the color change was monitored and was compared to negative control. Appropriate reagents were added as required for particular reaction as described in the kit manual. Finally, the species assignment was done using API software (APIweb™ API Staph V4.1).

Furthermore, to identify the source of airborne staphylococci contamination, a multiplex PCR of staphylococcal thermonuclease (*nuc*) gene targeting all human associated staphylococci species, i.e., *S. aureus, S. capitis, S. caprae, S. epidermidis, S. haemolyticus, S. hominis, S. lugdunensis, S. saprophyticus, and S. warneri* was also performed, using the primers and PCR conditions as described previously [24].

#### PCR based identification of *mecA* gene encoding for oxacillin resistance

The identification of *mecA* gene encoding for methicillin resistance in *Staphylococcus* species was done by PCR. The PCR reaction mixture was prepared by adding and mixing 2 μL template DNA 1 μL each primer (*mecA*) forward and reverse as described elsewhere [32], 2 μL each deoxynucleotide triphosphate (2.5 mM dNTP), 2.5 μL 10X Ex *Taq* buffer, and 0.5 μL Ex *Taq* polymerase (Takara Co., Ltd., Kyoto, Japan), in a final volume of 25 μL. A PCR thermal cycler was used for the final amplification of the PCR product. The reaction was set for amplification, with an initial denaturation step (95 °C, 3 min); 30 cycles of denaturation (95 °C, 30 s), annealing (47 °C, 45 s), and extension (72 °C, 30 s); and a final elongation step at 72 °C for 5 min. PCR products were visualized by electrophoresis in 1X Tris-acetate-EDTA on a 1% agarose gel stained with ethidium bromide. *S. aureus* ATCC 33591 and *S. aureus* ATCC 29213 was used as *mecA* positive and negative control, respectively.

## Data Availability

The datasets used and/or analysed during the current study are available from the corresponding author on reasonable request.

## Abbreviations

DBRACL: Dr. B.R. Ambedkar central library
CLAR: Central laboratory animal resources
MIC: Minimum inhibitory concentration
MRS: Methicillin resistant Staphylococci
CFU: Colony forming unit
CPS: Coagulase positive Staphylococci
CNS: Coagulase negative Staphylococci
GPB: Gram positive bacteria
GNB: Gram negative bacteria
MRSA: Methicillin resistant *Staphylococcus aureus*
MS-CNS: Methicillin sensitive- coagulase negative Staphylococci
MR-CNS: Methicillin resistant- coagulase negative Staphylococci
MDR-CNS: Multidrug resistant- coagulase negative Staphylococci
SSTIs: Skin and soft tissue infections
EMB: Eosin methylene blue
MSA: Mannitol salt agar
BA: Blood agar
BHI: Brain heart infusion
MHB: Mueller Hinton broth
HVAC: Heating, ventilation and air conditioning
